# Supplementary material for: Functional analysis of Cdc20 reveals a critical role of CRY box in mitotic checkpoint signaling
Source: Commun Biol. 2024 Feb 9;7:164. doi: 10.1038/s42003-024-05859-6 (PMC10858191; doi:10.1038/s42003-024-05859-6)
Supplement: Supplementary file 2 — Supplementary Information [file 42003_2024_5859_MOESM2_ESM.pdf]

Supporting Information

1. Supplementary Figures 1-5

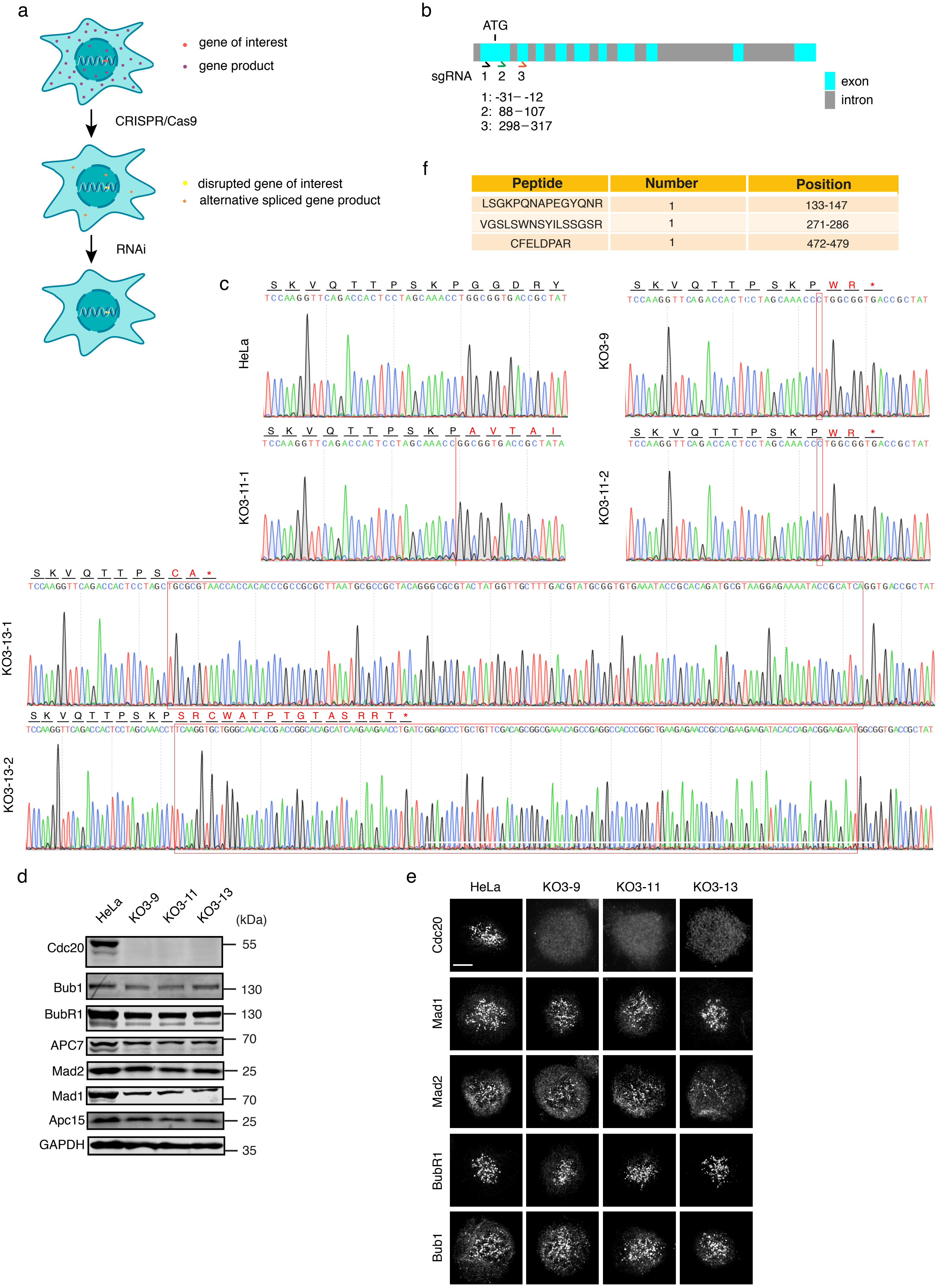

Peptide	Number	Position
LSGKPQNAPEGYQNR	1	133-147
VGSLSWNSYILSSGSR	1	271-286
CFELDPAR	1	472-479

Supplementary Figure 1 Characterization of *CDC20* knockout cell lines

a Cartoon showing the strategy of combining CRISPR/Cas9 and RNAi to achieve a clean background. b Schematic showing the target positions of three sgRNAs. Blue boxes indicate exons and gray ones are introns. Positions of genomic DNA recognized by each guide RNA were marked below the scheme. The number indicates the nucleotide position relative to A of ATG which was set to 1. c Genomic sequencing chromatography of parental cells and the knockout cells from sgRNA #3. Inserted nucleotides were marked by the red box and the position of deleted nucleotide was marked by the red line. Amino acids encoded were presented above the DNA sequence with red letters indicating the ones by shifted reading frame. Star means premature termination codon. KO3-9 has an extra C inserted; KO3-11 has either one T deleted (left) or an extra C inserted (right); KO3-13 has either 9 nucleotides deleted and 119 extra nucleotides inserted (above) or 137 extra nucleotides inserted (bottom). d Western blot analysis of the checkpoint proteins and APC/C proteins in knockout cells from sgRNA #3. Mitotic cells were collected and analyzed by quantitative western blot with corresponding antibodies. See Supplementary Fig. 5 for uncropped blots. e Kinetochore localization of checkpoint proteins in parental cells and knockout cells from sgRNA #3. Nocodazole (200 ng/ml) treated cells were fixed and stained by the corresponding antibodies. f Cdc20 peptides identified by mass spectrometry of the immunoprecipitate with Cdc20 antibody against C-terminal region from KO3-9 cells.

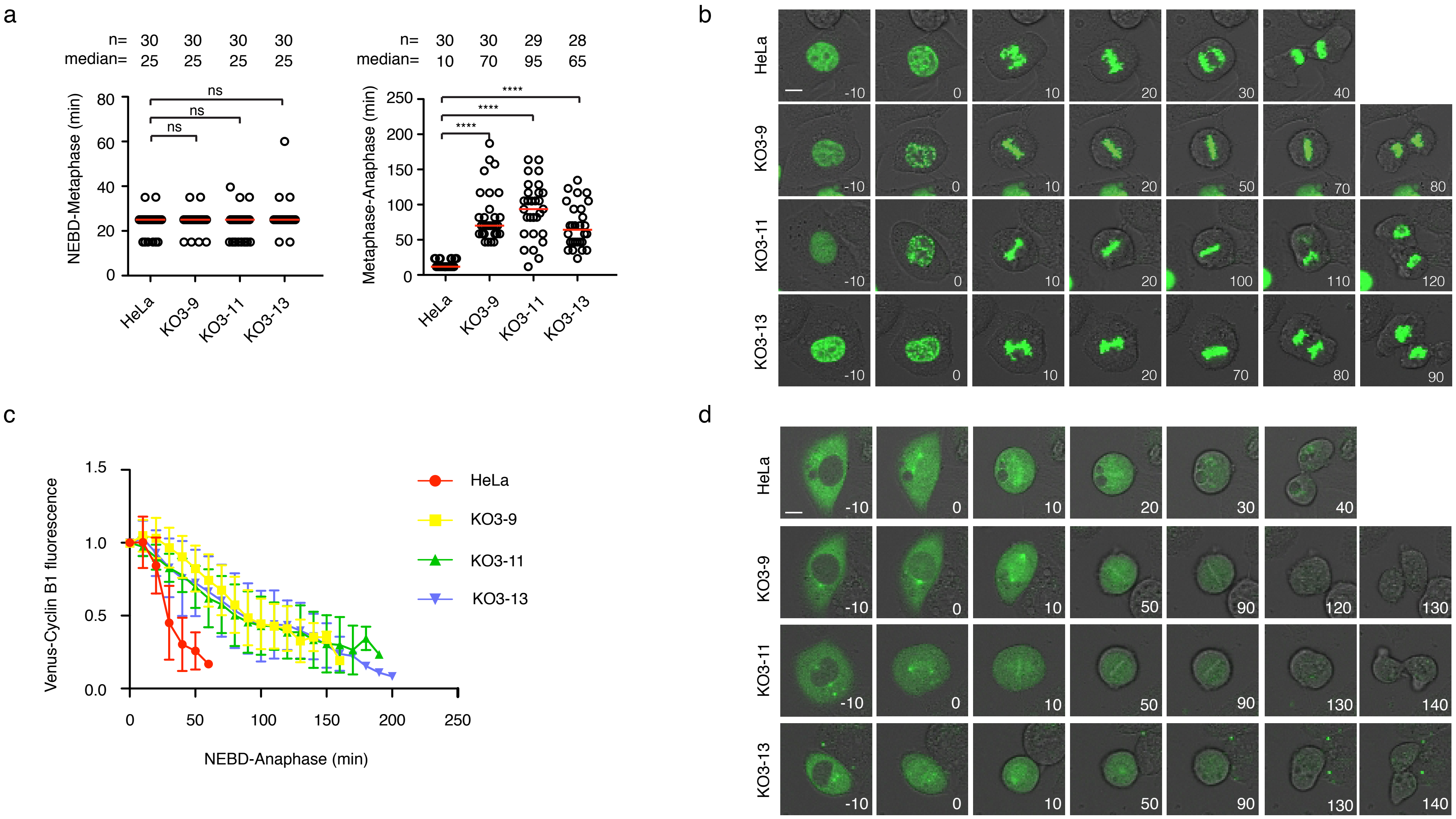
a
n= 30 30 30 30
median= 25 25 25 25
NEBD-Metaphase (min)
ns
ns
ns
HeLa
KO3-9
KO3-11
KO3-13
n= 30 30 29 28
median= 10 70 95 65
Metaphase-Anaphase (min)
****
****
****
HeLa
KO3-9
KO3-11
KO3-13
b
HeLa
KO3-9
KO3-11
KO3-13
c
Venus-Cyclin B1 fluorescence
NEBD-Anaphase (min)
HeLa
KO3-9
KO3-11
KO3-13
d
HeLa
KO3-9
KO3-11
KO3-13

Supplementary Figure 2 *Cdc20* knockout cells display a metaphase-anaphase transition delay due to slow degradation of Cyclin B1

a The mitotic progress was recorded in HeLa cells or *CDC20* knockout cells expressing YFP-H3. The time from NEBD to metaphase (left) and from metaphase to anaphase (right) were analyzed and presented separately. Each circle represents the time of a single cell and the red line indicates the median time. The number of cells analyzed per condition is indicated above (n = X). Representative experiment of two independent experiments is shown. Mann-Whitney u-test was applied. ns means not significant; **** means $P<0.0001$. b Representative stills of a. c Plot showing YFP-Cyclin B1 degradation from NEBD till anaphase in HeLa cells or *CDC20* knockout cells. The average intensity from 30 cells is presented on each time point. The intensity at time 0 is set to 1. Representative experiment of two independent experiments is shown. d Representative stills of c.

a

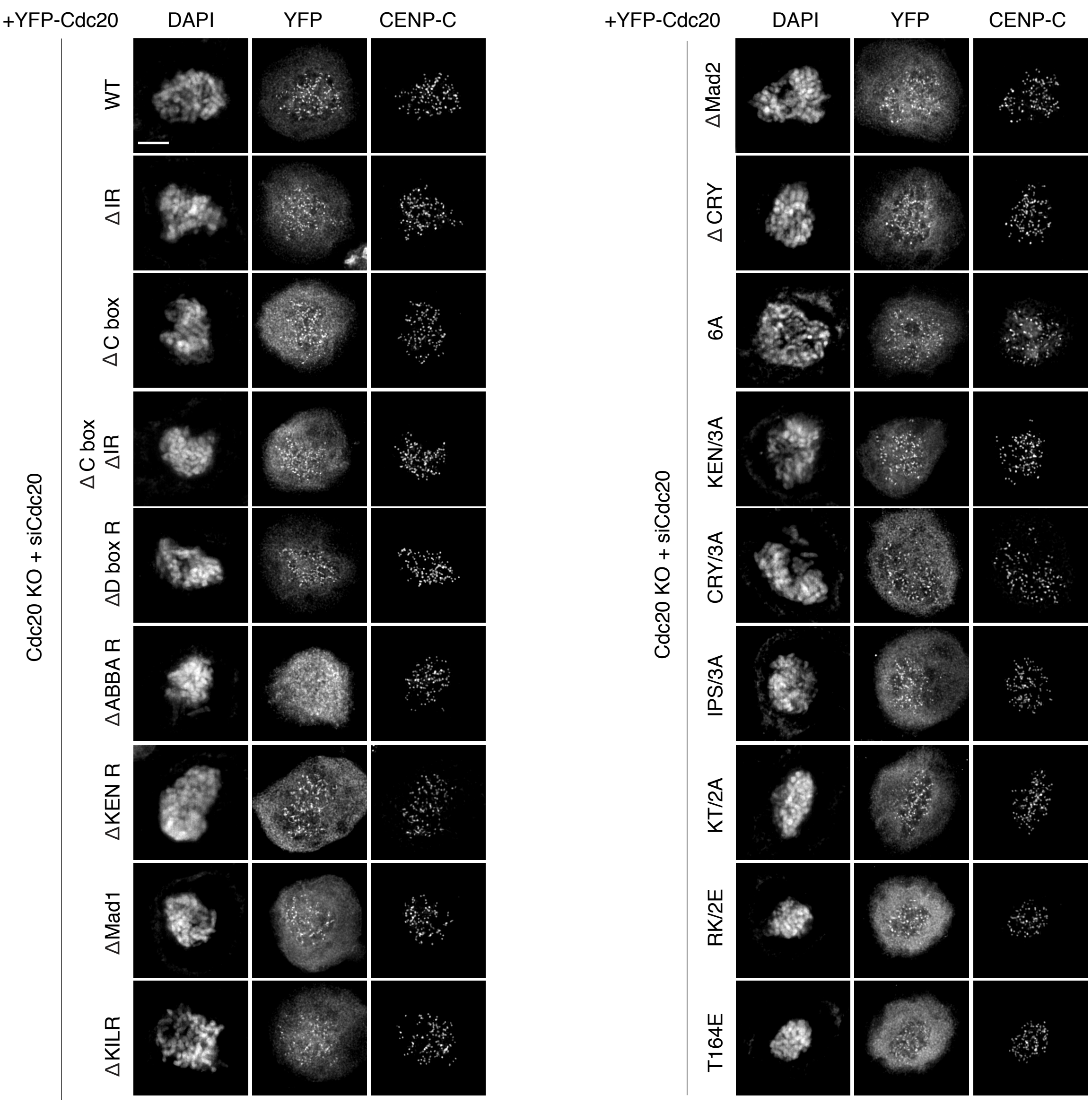
+YFP-Cdc20
DAPI
YFP
CENP-C
Cdc20 KO + siCdc20
WT
ΔIR
ΔC box
ΔC box ΔIR
ΔD box R
ΔABBA R
ΔKEN R
ΔMad1
ΔKILR
ΔMad2
ΔCRY
6A
KEN/3A
CRY/3A
IPS/3A
KT/2A
RK/2E
T164E

b

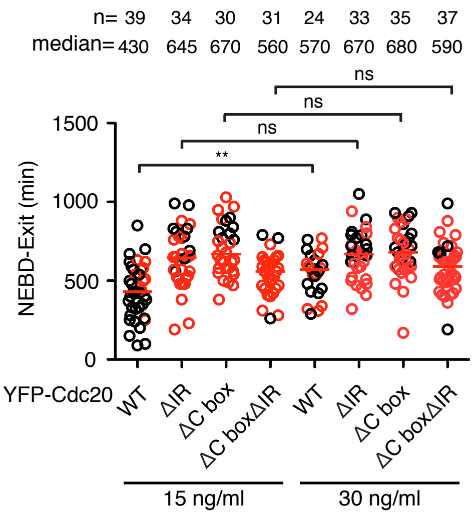
n= 39 34 30 31 24 33 35 37
median= 430 645 670 560 570 670 680 590
ns
ns
ns
**
NEBD-Exit (min)
1500
1000
500
0
YFP-Cdc20
WT
ΔIR
ΔC box
ΔC boxΔIR
WT
ΔIR
ΔC box
ΔC boxΔIR
15 ng/ml
30 ng/ml

c

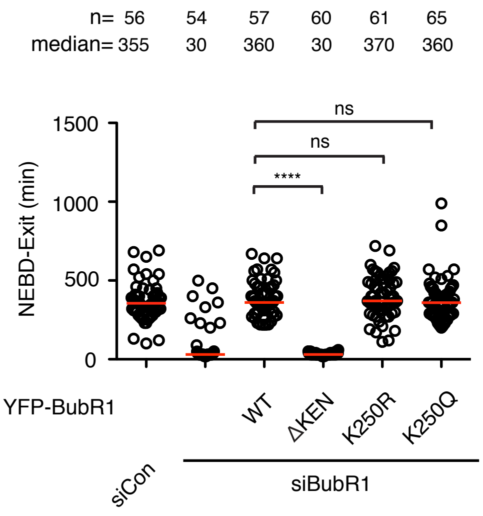
n= 56 54 57 60 61 65
median= 355 30 360 30 370 360
ns
ns
****
NEBD-Exit (min)
1500
1000
500
0
YFP-BubR1
WT
ΔKEN
K250R
K250Q
siCon
siBubR1

Supplementary Figure 3 Characterization of Cdc20 mutants on localization and checkpoint activity

a Knockout cells were transfected with YFP-Cdc20 constructs and siRNA oligos against Cdc20. Nocodazole (200 ng/ml) treated cells were fixed and stained with corresponding antibodies. b The time from NEBD to mitotic exit in the presence of nocodazole at two different concentrations (15 ng/ml or 30 ng/ml) of the knockout cells complemented with wild type or mutant YFP-Cdc20 and depleted the residual endogenous Cdc20 by RNAi. Red circle means cell died after mitotic arrest. c The time from NEBD to mitotic exit in the presence of nocodazole (30 ng/ml) of HeLa cells complemented with wild type or mutant YFP-BubR1 and depleted the endogenous BubR1 by RNAi. Each circle represents a single cell from NEBD to mitotic exit and the red line indicates the median time. The number of cells analyzed per condition is indicated above (n = X). Representative experiment of two independent experiments is shown. Mann-Whitney u-test was applied. ns means not significant; ** means $P<0.01$; **** means $P<0.0001$.

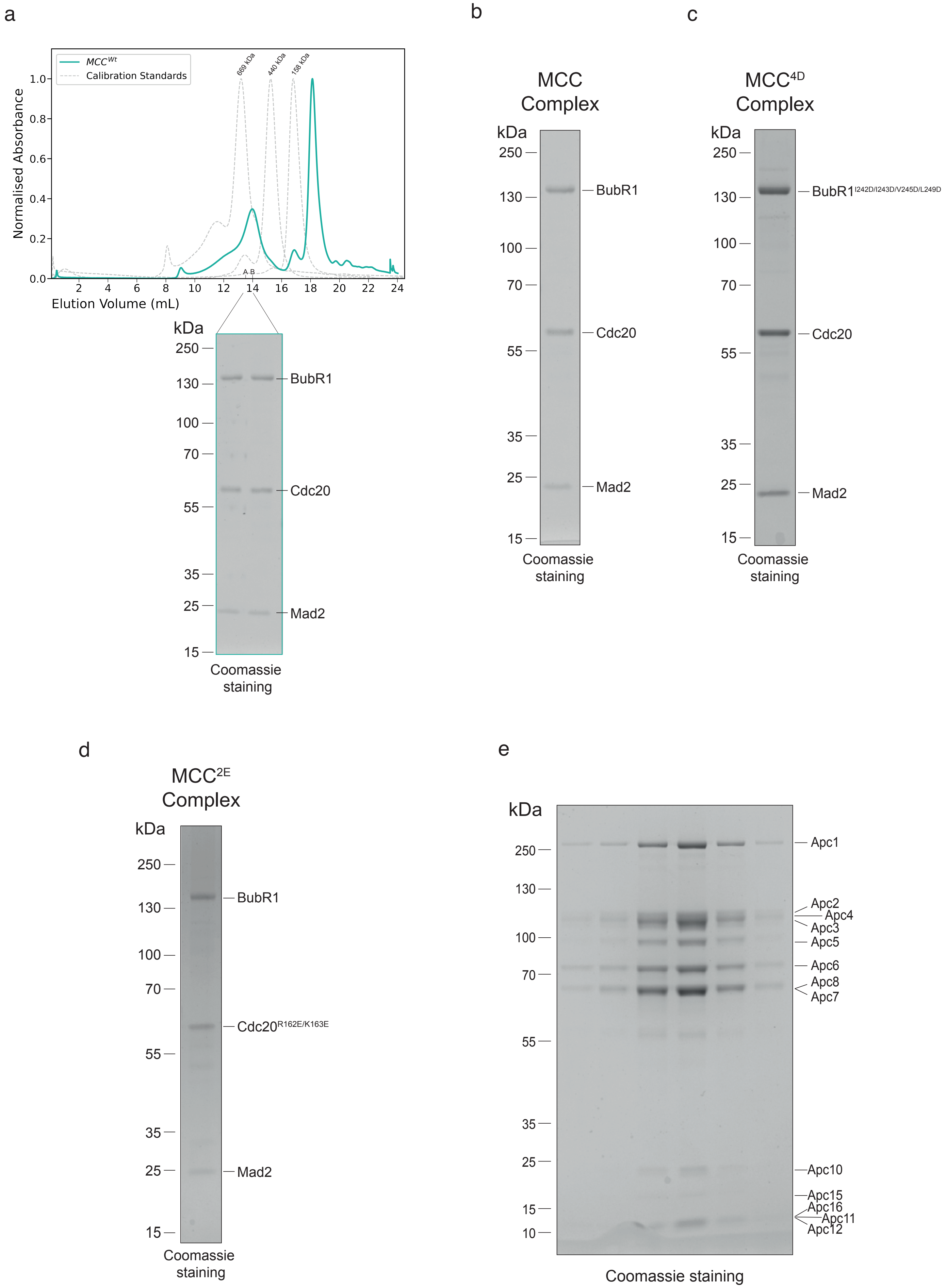

a
MCC^Wt
Calibration Standards
669 kDa
440 kDa
158 kDa
Normalised Absorbance
1.0
0.8
0.6
0.4
0.2
0.0
A B
2 4 6 8 10 12 14 16 18 20 22 24
Elution Volume (mL)
kDa
250
130
100
70
55
35
25
15
BubR1
Cdc20
Mad2
Coomassie staining
b
MCC Complex
kDa
250
130
100
70
55
35
25
15
BubR1
Cdc20
Mad2
Coomassie staining
c
MCC4D Complex
kDa
250
130
100
70
55
35
25
15
BubR1 I242D/I243D/V245D/L249D
Cdc20
Mad2
Coomassie staining
d
MCC2E Complex
kDa
250
130
100
70
55
35
25
15
BubR1
Cdc20 R162E/K163E
Mad2
Coomassie staining
e
kDa
250
130
100
70
55
35
25
15
10
Apc1
Apc2
Apc4
Apc3
Apc5
Apc6
Apc8
Apc7
Apc10
Apc15
Apc16
Apc11
Apc12
Coomassie staining

Supplementary Figure 4 Reconstitution of MCC and APC/C

Biochemical reconstitutions of the MCC complexes and the APC/C. a A chromatogram of MCC^{Wt} from size exclusion chromatography (top). The calibration standards are shown in grey. MCC^{Wt} elutes between 12 and 16 mL. The second peak at 18.5 mL accounts for both 3C-GST and tev used to cleave tags from all three subunits. Coomassie stained gel of the eluted fractions (bottom). b-d SDS-PAGE of the reconstituted MCC complexes used in this study. e) SDS-PAGE of the reconstituted APC/C. See Supplementary Fig. 5 for uncropped blots.

Fig 1a

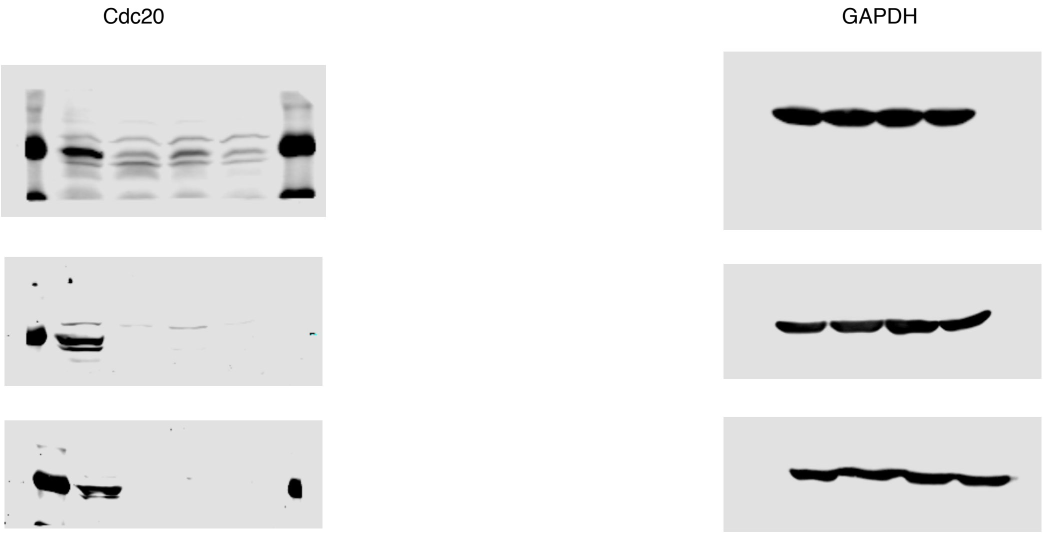

Fig 4e

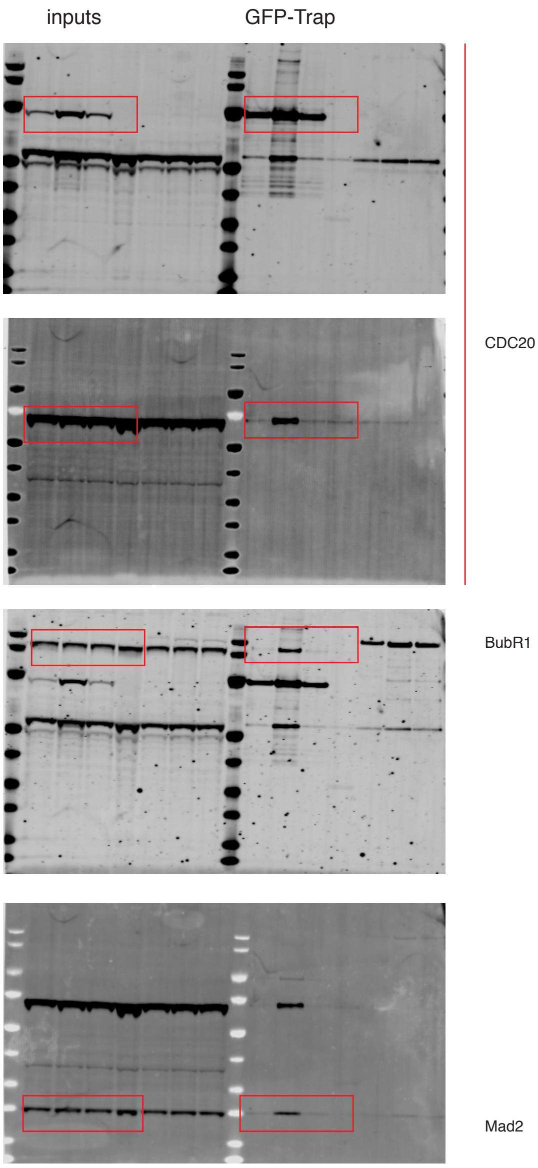

Fig 4f

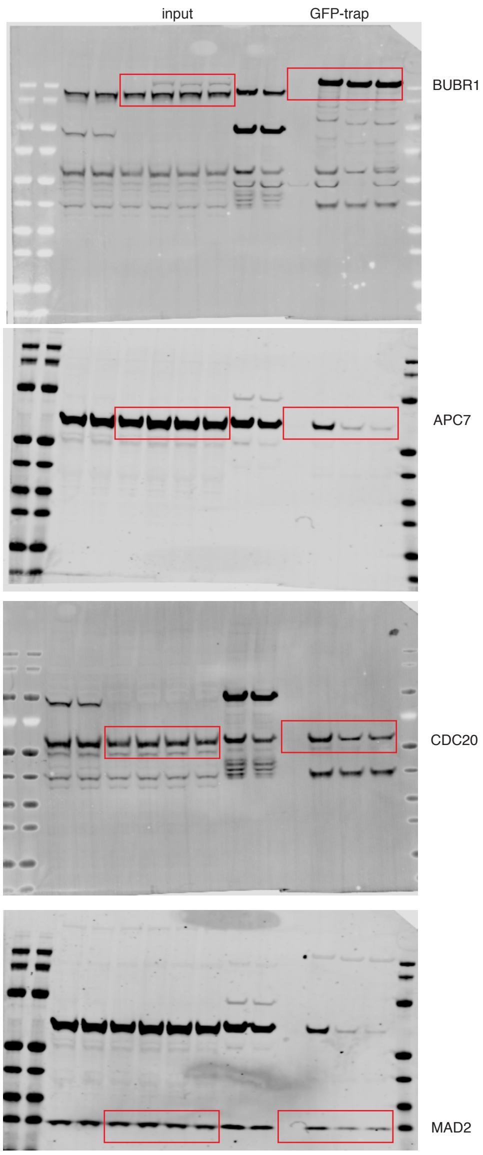

Fig 4g

Repeat 1

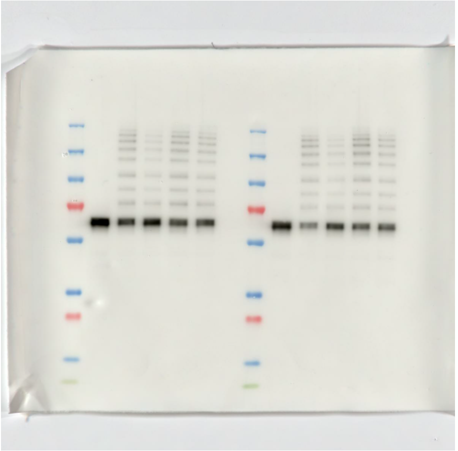

Repeat 2

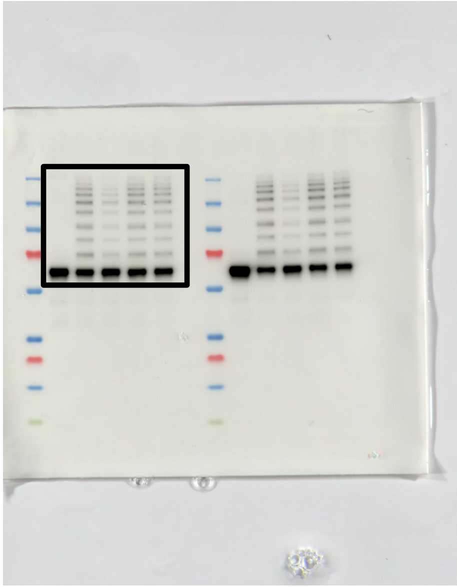

Repeat 3

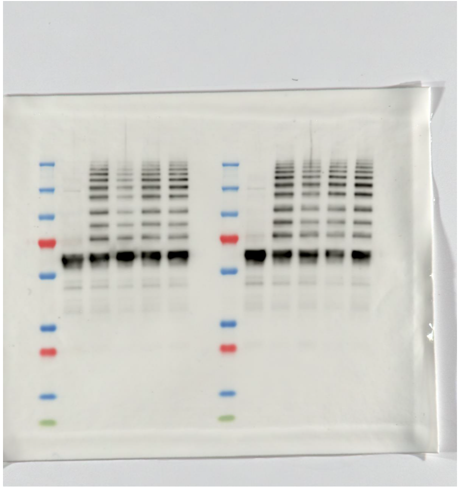

Supplementary Fig 1d

Cdc20

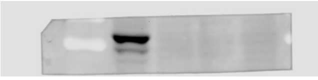

Bub1

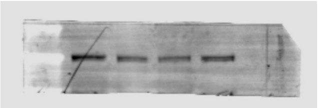

BubR1

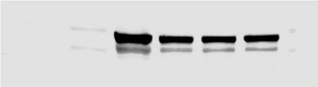

Apc7

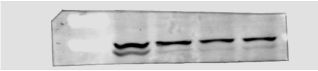

Mad2

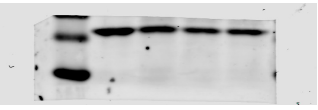

Mad1

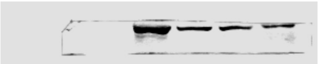

Apc15

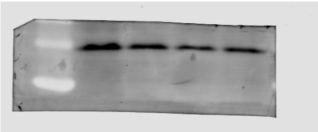

GAPDH

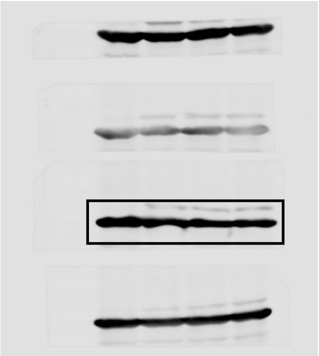

Supplementary Fig 4a

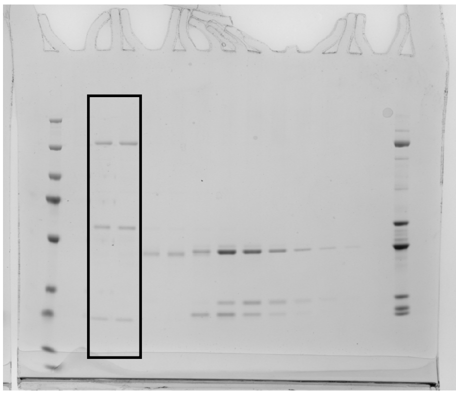

Supplementary Fig 4d

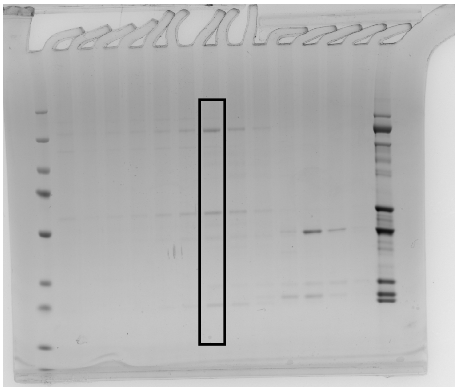

Supplementary Fig 4b

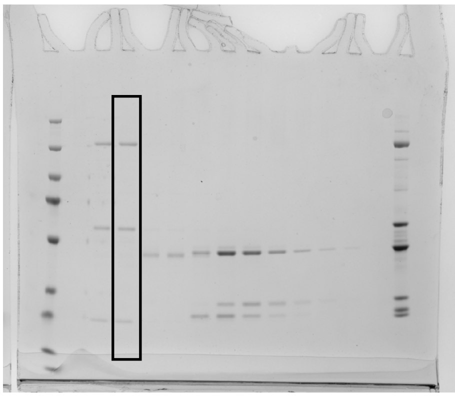

Supplementary Fig 4e

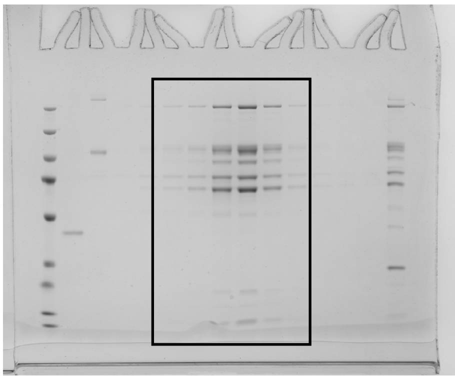

Supplementary Fig 4c

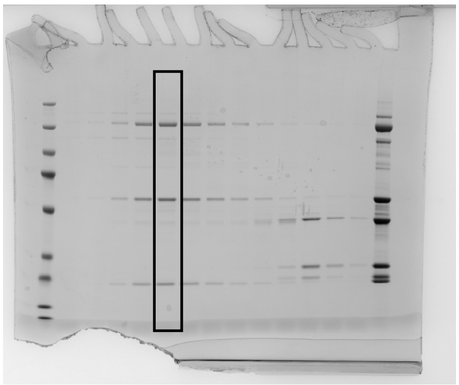

Supplementary Figure 5 All uncropped Blots

The above were all uncropped blots for Figures and Supplementary Figures in the main text.
